# Comparative Transcriptome Analysis Reveals Mechanisms of Folate Accumulation in Maize Grains

**DOI:** 10.3390/ijms23031708

**Published:** 2022-02-01

**Authors:** Tong Lian, Xuxia Wang, Sha Li, Haiyang Jiang, Chunyi Zhang, Huan Wang, Ling Jiang

**Affiliations:** 1 Biotechnology Research Institute, Chinese Academy of Agricultural Sciences, Beijing 100081, China; liantong9111@163.com (T.L.); lisha2012@nwsuaf.edu.cn (S.L.); zhangchunyi@caas.cn (C.Z.); 2 Plant Genetics, Gembloux Agro-Bio Tech, University of Liège, 5030 Gembloux, Belgium; 3 Sanya Institute, Hainan Academy of Agricultural Sciences, Sanya 572000, China; 4 School of Life Sciences, Anhui Agricultural University, Hefei 230036, China; 17720423@stu.ahau.edu.cn (X.W.); hyjiang@ahau.edu.cn (H.J.); 5 National Agricultural Science and Technology Center, Chengdu 610213, China

**Keywords:** folate accumulation, transcriptome, WGCNA, maize kernel

## Abstract

Previously, the complexity of folate accumulation in the early stages of maize kernel development has been reported, but the mechanisms of folate accumulation are unclear. Two maize inbred lines, DAN3130 and JI63, with different patterns of folate accumulation and different total folate contents in mature kernels were used to investigate the transcriptional regulation of folate metabolism during late stages of kernel formation by comparative transcriptome analysis. The folate accumulation during DAP 24 to mature kernels could be controlled by circumjacent pathways of folate biosynthesis, such as pyruvate metabolism, glutamate metabolism, and serine/glycine metabolism. In addition, the folate variation between these two inbred lines was related to those genes among folate metabolism, such as genes in the pteridine branch, para-aminobenzoate branch, serine/tetrahydrofolate (THF)/5-methyltetrahydrofolate cycle, and the conversion of THF monoglutamate to THF polyglutamate. The findings provided insight into folate accumulation mechanisms during maize kernel formation to promote folate biofortification.

## 1. Introduction

Folate, a water-soluble vitamin B9, acts as the one-carbon acceptor and donor involved in the biosynthesis of amino acids, nucleic acids, proteins, and lipids [1]. In plants, folate can be synthesized de novo, which makes plants serve as a good folate source for humans. However, folate contents in edible parts of crops, such as maize (*Zea mays*), rice (*Oryza sativa*), and potato (*Solanum tuberosum*), are at low levels compared with green vegetables [2,3,4]. Folate deficiency increases the risk of neural tube defects and always is a global public health issue [5,6]. Folate biofortification by metabolic engineering of crops, e.g., maize, rice, potato, tomato (*Solanum lycopersicum*) and wheat (*Triticum aestivum*), have been investigated to obtain folate-enriched food to alleviate this problem [2]. In plants, the connection between folate metabolism and other metabolites has been explored to understand the mechanism of folate accumulation. Most vitamins have the same upstream precursors from the pentose and triose pool, which produce chorismate for the biosynthesis of tocochromanols, phylloquinone, and folates, and ribose 5-P for the biosynthesis of thiamine, riboflavin, and folates [7,8,9,10,11]. Furthermore, glutamine and glutamate, which are involved in a set of reactions within the specific and shared biosynthetic pathways of B vitamins and purine, connects to thiamine, riboflavin, and folates [10]. In addition, the dysfunctions of folate metabolism impact the balance of NADPH/NADP ratio, nitrogen metabolism, and photorespiration in plants [12,13,14,15,16,17]. These complexities indicate that plant folate accumulation in plants could be affected by multiple elements and environmental cues.

The folate biosynthesis pathway has been well described in plants [18]. The upstream precursors for folate mainly come from carbohydrate metabolism, which provides erythrose-P and phosphoenolpyruvate for chorismate synthesis, and ribose 5-P for GTP synthesis [10,11]. Chorismate can be converted to para-aminobenzoate (ρ-ABA) by aminodeoxychorismate synthase (ADCS) and aminodeoxychorismate lyase (ADCL) in plastids. Moreover, GTP can be converted to 6-hydroxymethyldihydropterin (HMDHP) by GTP cyclohydrolase I (GCHI) and dihydroneopterin aldolase (DHNA) in the cytosol. Subsequently, the HMDHP and ρ-ABA are assembled into dihydrofolate (DHF) by HMDHP pyrophosphokinase/dihydropteroate synthase (HPPK/DHPS) and dihydrofolate synthetase (DHFS) in the mitochondrion. Later, bifunctional dihydrofolate reductase-thymidylate synthase (DHFR-TS, DRTS) catalyzes the DHF to tetrahydrofolate (THF). Then, THF can be polyglutamylated through folylpolyglutamate synthetase (FPGS). Afterwards, the glutamate tail can be removed by γ-glutamyl hydrolase (GGH) [19]. In one-carbon metabolism, one-carbon units, which derive from polyglutamylated THF, can be generated directly within the conversion of THF and serine to glycine and 5,10-methylene-THF (5,10-CH=THF) by serine hydroxymethyltransferase (SHMT) and glycine cleavage system (GCS). Later, the conversion among different folate derivatives responsible for the biosynthesis of purine, thymidylate (dTMP), and methionine by dehydrogenase/5,10-CH=THF cyclohydrolase (DHC), 10-formyl THF deformylase (10-FDF), 10-formyltetrahydrofolate synthetase (FTHS), methylenetetrahydrofolate reductase (MTHFR), methionine synthesis (MS), and 5-formyl THF cycloligase (5-FCL) [20,21]. In plants, several folate biosynthetic genes are developmentally regulated. For example, leaves, roots, and stems express the HPPK-DHPS enzyme during seedling development in pea (*Pisum sativum*) [22]. In tomatoes, *GCHI* transcript and protein level are expressed strongly in unripe fruit but not ripe fruit. The *ADCS* and *ADCL* transcript levels in mature green fruits were approximately 10% in leaves; then, they decreased to nothing after the breaker stage [23,24]. Otherwise, some others are subject to feedforward control by pathway intermediates [25]. For instance, both plant ADCS and DHPS are inhibited by the downstream products DHP and DHF in vitro [9,26] and overexpressing exogenous *GCHI* and *ADCS* in tomato fruit; the transcription levels of the respective objective genes do not change, whereas those of the downstream genes *ADCL*, *DHNA,* and mitochondrial *FPGS* are increased [25]. Indeed, the transcript expression levels of several folate biosynthesis genes were associated with the folate contents, such as *GGH1* in potato [27] as well as *GCHI* and the *ADCS* enzyme in *pak choi* [28]. Nevertheless, the transcriptional regulation of plant folate metabolism still needs more exploration.

Maize is a significant source of food, feed, and industrial products [29]. The complexity of folate accumulation in the early stages of maize kernel development has been reported, but mechanisms of folate accumulation have not been well revealed [30]. In this study, transcriptional levels of the folate-related genes in low- and high folate maize inbred lines during the late stages of kernel formation were investigated by RNA-seq analysis. Comparative transcriptome analysis and weighted gene co-expression network analysis (WGCNA) were performed to reveal how folates accumulate in maize kernel. The results showed that folate accumulation during the late stages of kernel development might be mostly related to pyruvate metabolism, glutamate metabolism, and serine/lysine metabolism. Additionally, the variation of folate concentration between different inbred lines may be relevant to those genes belonging to the pteridine branch, ρ-ABA branch, serine/THF/5-methyltetrahydrofolate (5-M-THF) cycle, and conversion of monoglutamate THF to polyglutamate THF. The findings provided valuable knowledge of the folate accumulation mechanism during maize kernel formation for further folate biofortification and breeding in the future.

## 2. Results

### 2.1. Folate Metabolic Profiling during Late Developmental Stages of Kernels

**Figure 1:**
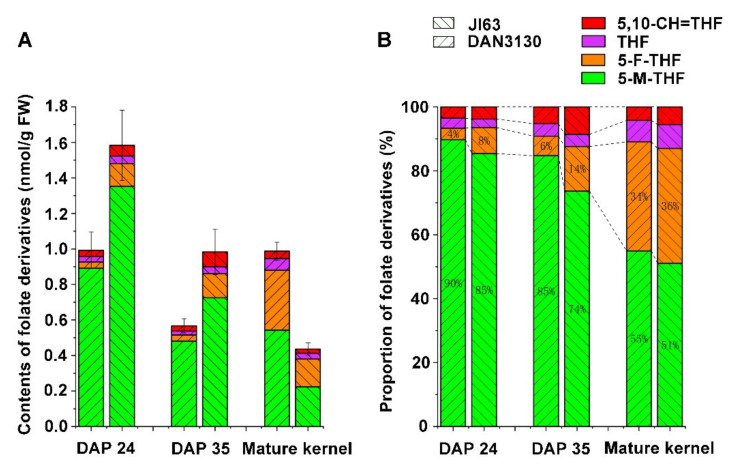
Folate profiling of maize kernel during late stages of development. (**A**) contents of folate derivatives on DAP 24, DAP 35, and mature kernels in DAN3130 and JI63. (**B**) the proportion of different folate derivatives. DAP, the day after pollination; 5-M-THF, 5-methyl-tetrahydrofolate; 5-F-THF, 5-formyl-tetrahydrofolate; 5,10-CH=THF, 5,10-methylene-tetrahydrofolate; THF, tetrahydrofolate.

Two maize inbred lines DAN3130 and JI63 had a significant difference in total folate content in mature kernels. Four kinds of folate derivatives were detected by liquid chromatography-tandem mass spectroscopy (LC/MS), including 5-M-THF, 5-formyltetrahydrofolate (5-F-THF), 5,10-CH=THF, and THF. At the same harvest stage (mature kernel), the total folate content of DAN3130 was 2.25-fold that of JI63 (0.99 ± 0.02 nmol/g DW versus 0.44 ± 0.02 nmol/g DW), and their differences were attributed to the inbred-specific variation (Figure 1A, Table S1). In contrast, they shared a similar proportion of folate derivatives (5-M-THF, 55% versus 51%; 5-F-THF, 34% versus 36%; 5,10-CH=THF, 6.7% versus 7.5%; THF, 4.2% versus 5.5%) (Figure 1B), indicating that 5-M-THF and 5-F-THF were the major folate derivatives in mature kernels.

To investigate the folate accumulation pattern during the late stage of kernel formation in maize, the immature kernels on the day after pollination (DAP) 24 and DAP 35 were collected for folate analysis. Interestingly, even though DAN3130 had higher levels of folates in mature kernels compared to JI63, its folate contents on DAP 24 and DAP 35 were significantly lower than JI63 (Figure 1A). Considering the patterns of folate accumulation from DAP 24 to DAP 35, both inbred lines had a similar accumulation pattern. The level of 5-F-THF was stable, but 5-M-THF decreased significantly (Figure 1A). Whereas from DAP 35 to mature kernels, in DAN3130, the levels of 5-F-THF sharply increased while 5-M-THF was unchanged, resulting as the increase in total folates to 0.99 ± 0.05 nmol/g. In JI63, levels of 5-M-THF decreased significantly, and 5-F-THF was stable, resulting as the decrease in total folate to 0.44 ± 0.02 nmol/g (Figure 1A). From DAP 24 to mature kernels, the proportion of THF and 5-F-THF increased significantly while 5-M-THF decreased in both lines (Figure 1B). Then, the dynamic patterns of folate derivatives from DAP 24 to mature kernel were regarded as the development-specific variation. Thus, the development-specific variation and inbred-specific variation were further analyzed by using comparative transcriptomes to investigate the critical roles associated with folate accumulation during kernel formation.

### 2.2. Transcriptome Profiling of DAN3130 and JI63 during Kernel Development by RNA-Seq

**Figure 2:**
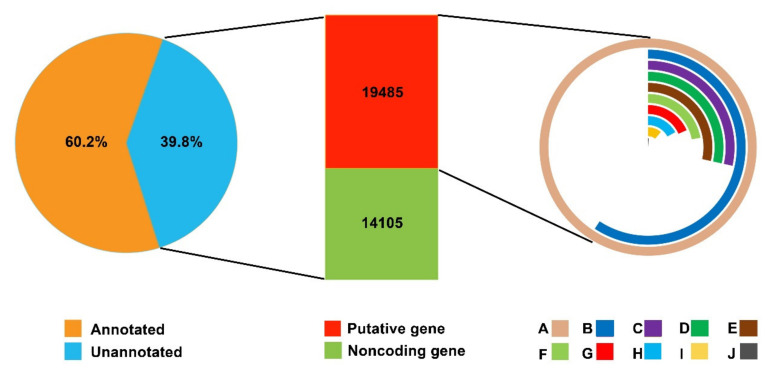
RNA-seq analysis of two inbred lines and annotation source. A: total numbers of genes; B: homologous analysis of DNA sequence (nr); C: SignalP prediction; D: TMHMM prediction; E: amino acid sequence prediction; F: homologous analysis of amino acid sequence; G: homologous analysis of DNA sequence (UniProt); H: PFAM prediction; I: homologous analysis of amino acid sequence (UniProt); J: RNA MMER identification.

DAN3130 and JI63 inbred lines were grown in Langfang, China. The fresh kernel samples of each inbred line were collected on DAP 24 and DAP 35, respectively, and then frozen in liquid nitrogen immediately. Mature kernel samples were harvested after all the plants turned yellow. Three biological replicates of each sample were collected, and total RNA with high quality was pooled and sent for sequencing. Total RNA of high quality was pooled for transcriptome analysis, and raw RNA-seq data of DAP 24, DAP35, and mature kernels for both two inbred lines were obtained (Table S2). All reads were aligned to the B73 genome, and most of the reads matched the maize genome with a unique location. The quality and accuracy of the sequencing data were sufficient for further analysis. All assembled transcripts were compared to genes in the NCBI non-redundant nucleotide (nt) database using BLASTn, and 50,810 unigenes with similarity to known genes were annotated. Then, those unannotated unigenes were searched against the Swiss-Prot and NCBI non-redundant protein (nr) databases using Trinoate with an E-value cutoff of 1 × 10^−3^. Among the remaining 33,590 unigenes, 19,485 were annotated to known genes/proteins, and 14,105 were identified as non-protein-coding genes (Figure 2).

### 2.3. Screening of Differentially Expressed Genes (DEGs) in the Development-Specific Variation

To figure out the relationship between the folate accumulation and other metabolism pathways in maize, comparisons between transcriptomes of DAN3130 and JI63 were performed, and seven different comparisons were analyzed: for the development-specific variation during development in the same line (I: DAN3130 DAP 24 versus DAN3130 DAP 35, II: JI63 DAP 24 versus JI63 DAP 35, III: DAN3130 DAP 35 versus DAN3130 mature kernels, IV: JI63 DAP 35 versus JI63 mature kernels) and for the inbred-specific variation between two lines at the same time point (DAN3130 DAP 24 versus JI63 DAP 24, DAN3130 DAP 35 versus JI63 DAP 35, DAN3130 mature kernels versus JI63 mature kernels).

**Figure 3:**
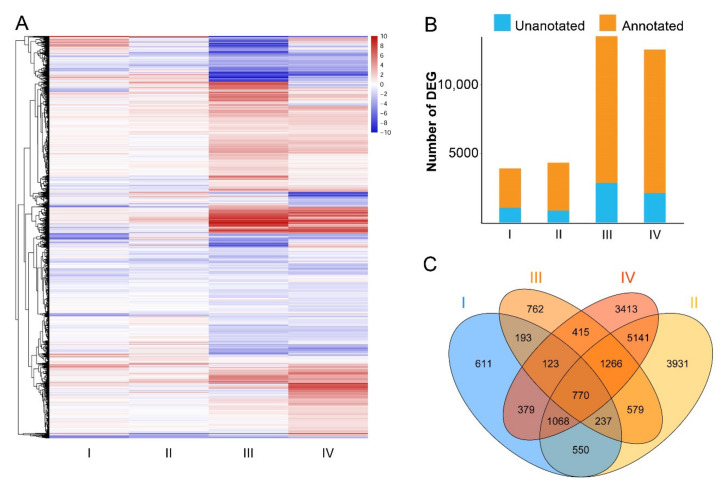
Screening of DEGs in development-specific variation; (**A**) heatmap of DEGs in comparison between different stages; (**B**) number of DEGs in comparison between different stages; (**C**) Venn diagram of unique and common DEGs in comparison between different stages. I, DAN3130 DAP 24 versus DAP 35; II, JI63 DAP 24 versus DAP 35; III, DAN3130 DAP 35 versus mature kernel; IV, JI63 DAP 35 versus mature kernel.

Since different folate derivatives had different accumulation patterns during late stages of kernel development, the comparisons of DAP 24 versus DAP 35 and DAP 35 versus mature kernels in the same inbred line were analyzed to figure out the transcriptional variation with folate change during kernel development. The results showed that the variation level in gene expression of DAP 35 versus mature kernels was more observable than those in DAP 24 versus DAP 35 (Figure 3A). The numbers of DEGs during DAP 35 to mature kernels (13,542 (III) and 12,575 (IV) DEGs) was much more than DAP 24 to DAP 35 (3,931 (I) and 4,345 (II) DEGs) (Figure 3B), indicating that the variational gene expression of DAP 35 versus mature kernels was more complicated than DAP 24 versus DAP 35. Furthermore, 770 common genes were differentially expressed among the comparisons of I, II, III, and IV (Figure 3C).

### 2.4. Gene Ontology (GO) Enrichment and Kyoto Encyclopedia of Genes and Genomes (KEGG) Analysis of DEGs in Development-Specific Variation

**Figure 4:**
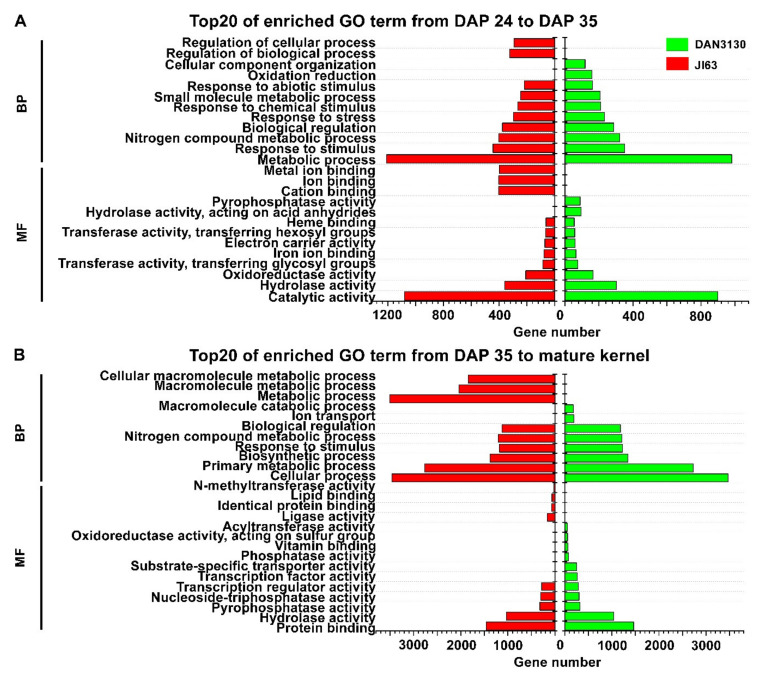
Bioinformatics analysis of DEGs in development-specific variation. (**A**) GO function analysis of DEGs during DAP 24 to DAP 35; (**B**) GO function analysis of DEGs during DAP 35 to mature kernel. MF, molecular function; BP, biological process.

GO and KEGG enrichment analyses were carried out further to study the function of DEGs in development-specific variation. The DEGs of Comparison I and Comparison III enriched in DAN3130 were assigned to 343 and 552 GO terms (*p*-value < 0.05), respectively. For JI63, the total 333 GO terms (*p*-value < 0.05) were observed in Comparison II and 533 GO terms (*p*-value < 0.05) were assigned to Comparison IV. The top 20 GO terms were screened out based on enriched gene numbers (Figure 4). Considering the similar accumulation pattern that occurred from DAP 24 to DAP 35, it is noteworthy that common GO terms such as catalytic activity, hydrolase activity, oxidoreductase activity, metabolic process, response to stimulus, and nitrogen metabolic process were significantly enriched from DAP 24 to DAP 35 in DAN3130 and JI63 (Figure 4A). Since the folate phenotype results showed different accumulation patterns of 5-M-THF and 5-F-THF from DAP 35 to mature kernel, we focused on the specific terms of *N*-methyltransferase activity and acyltransferase activity in JI63 and DAN3130, respectively (Figure 4B).

**Figure 5:**
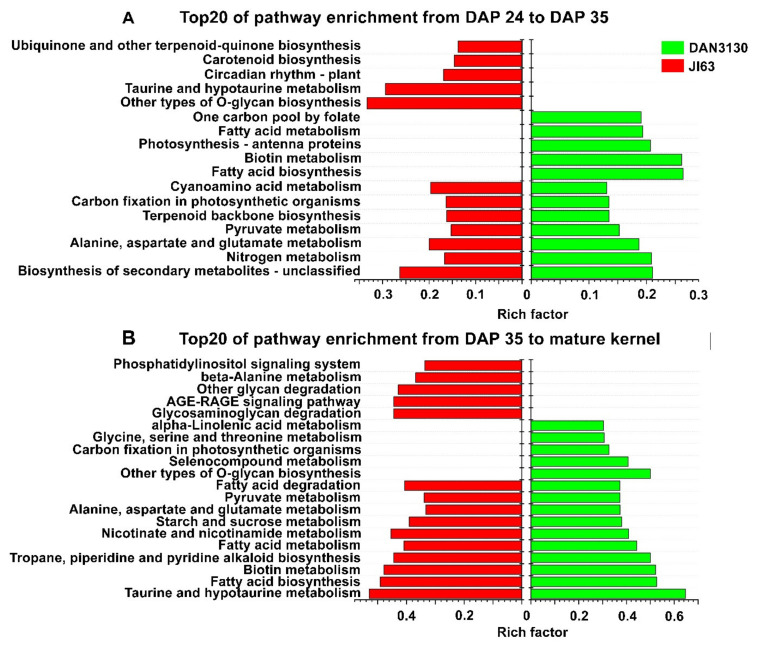
Bioinformatics analysis of DEGs in development-specific variation. (**A**) KEGG enrichment of DEGs during DAP 24 to DAP 35; (**B**) KEGG enrichment of DEGs during DAP 35 to mature kernel.

KEGG enrichment results showed DEGs from Comparison I and Comparison III assigned to 43 and 49 KEGG pathways (*p*-value < 0.05) in DAN3130, respectively. For JI63, a total of 31 and 52 KEGG pathways (*p*-value < 0.05) were identified from Comparison II and Comparison IV, respectively. The top 20 KEGG pathways based on the rich factor among the comparisons were screened out (Figure 5). The common pathways such as biosynthesis of secondary metabolites, nitrogen metabolism, glutamate metabolism and pyruvate metabolism were obviously enriched from DAP 24 to DAP 35, while one-carbon pool by folate pathway was explicitly found in DAN3130 (Figure 5A). From DAP 35 to mature kernel, the pathway of glutamate metabolism and pyruvate metabolism were identified again in both lines, while the glycine/serine metabolism pathway, which might be relevant to folate metabolism, was only found on DAN3130 (Figure 5B). Therefore, the folate variation during DAP 24 to mature kernel might be affected by the circumjacent pathways of folate biosynthesis such as pyruvate metabolism, glutamate metabolism, and glycine/serine metabolism, and also, those genes serve a molecular function for methyltransferase activity and acyltransferase activity.

### 2.5. Screening of DEGs in Inbred-Specific Variation

**Figure 6:**
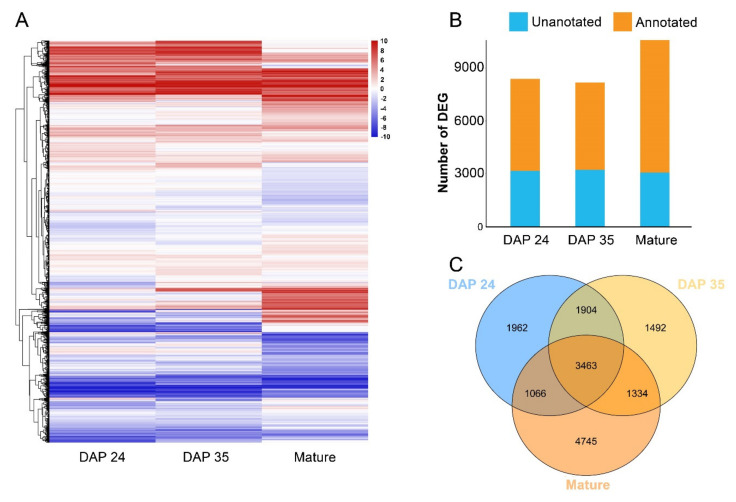
Screening of DEGs in the inbred-specific variation; (**A**) Heatmap of DEGs in comparison between line DAN3130 and JI63 on DAP 24, DAP 35, and mature kernels; (**B**) Number of DEGs in comparison between line DAN3130 and JI63 on DAP 24, DAP 35, and mature kernels; (**C**) Venn diagram of unique and common DEGs in the comparison between line DAN3130 and JI63 on DAP 24, DAP 35, and mature kernels.

Folate contents varied according to not only the development stages but also genotypes within species. Considering that significant differences in total folate contents were observed at the same harvest time point of these two lines, the comparison between two lines at the same harvest was summarized. A heatmap of DEGs in these comparisons showed that some genes had variational expression between two lines on mature kernels compared to DAP 24 and DAP 35 (Figure 6A). A total of 8395, 8193, and 10,608 DEGs on DAP 24, DAP 35, and the mature kernel were identified, respectively, indicating that the variation of transcriptional levels in mature kernels was more complex than in the immature stage between two lines (Figure 6B). In all, 3463 DEGs were commonly regulated between these two inbred lines from the Venn diagram (Figure 6C).

### 2.6. GO Enrichment and KEGG Analysis of DEGs in the Inbred-Specific Variation

**Figure 7:**
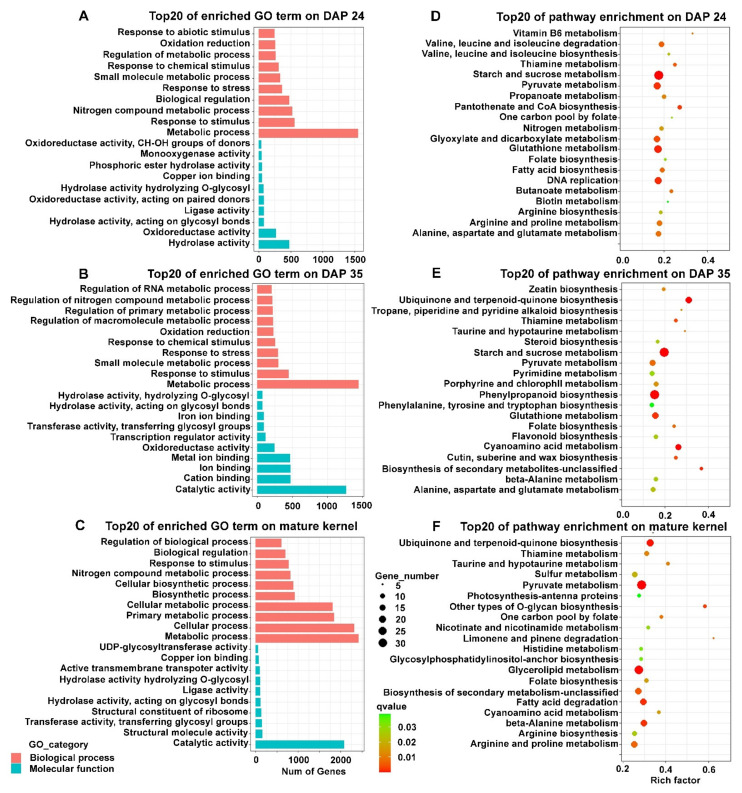
Bioinformatics analysis of DEGs in inbred-specific variation. (**A**–**C**) GO function analysis of DEGs on DAP 24, DAP 35, and mature kernel; (**D**–**F**) KEGG enrichment of DEGs on DAP 24, DAP 35, and mature kernel.

Based on the GO results, the DEGs of DAN3130 versus JI63 enrichment were assigned 251 GO terms (*p*-value < 0.05), 225 GO terms (*p*-value < 0.05), and 358 GO terms (*p*-value < 0.05) in DAP 24, DAP 35, and mature kernels, respectively. The top 20 GO terms were screened out based on enriched gene numbers, and the results displayed that the metabolic process was significantly found in all three stages in the biological process. From molecular function, the largest numbers of genes involved in catalytic activity were obviously found in DAP 35 and mature kernels while not in DAP 24 (Figure 7A–C).

The DEGs from DAN3130 versus JI63 on DAP 24, DAP 35, and mature kernels belonged to 36, 28, and 51 pathways (*p*-value < 0.05), respectively. The top 20 KEGG pathways based on rich factor were screened out in Figure 7D–F. Consistent with the KEGG result of development-specific variation, glutamate metabolism and pyruvate metabolism were identified again in inbred-specific variation. Unlike development-specific variation, the KEGG results of the inbred-specific variation at DAP 24, DAP 35, and mature kernel, respectively, all contain folate metabolism pathways, including folate biosynthesis and one-carbon pool by folate, indicating that the folate-related metabolism pathway might have contributions to folate variation of different inbred lines. In addition, several pathways indirectly involved in folate, such as vitamin B6 metabolism and thiamine metabolism, were also found. These results further demonstrated that folate biosynthesis and the one-carbon metabolism pathway could be tightly associated with folate variation of different inbred lines.

### 2.7. WGCNA Analysis

**Figure 8:**
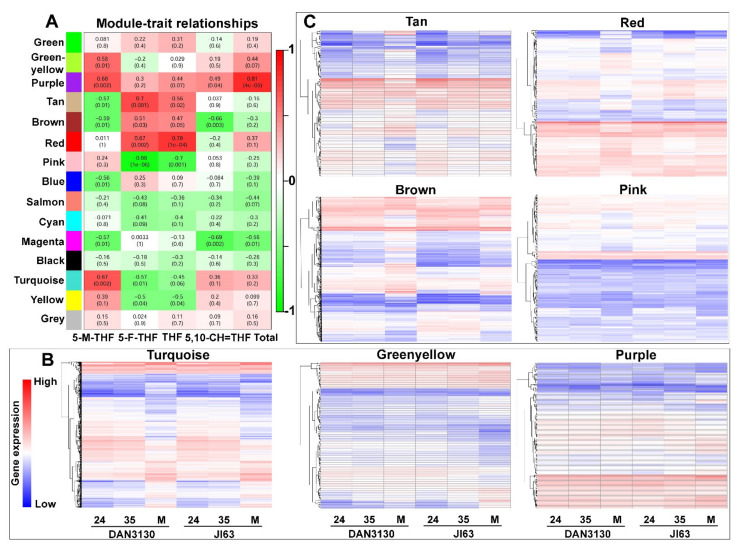
WGCNA analysis of kernel formation. (**A**) Heatmap of the correlation between modules and folate derivatives (each cell contained the correlation coefficient and corresponding *p*-value). (**B**) Gene expression trends of modules related to 5-M-THF; (**C**) Gene expression trends of modules related to 5-F-THF; 24, DAP 24; 35, DAP 35; M, mature kernel.

To systematically understand the gene sets related to the biological processes of folate accumulation, the WGCNA method was used to evaluate the gene expression network during kernel formation. The sample dendrogram and trait heatmap are shown in Figure S1A. The soft thresholding powers with β = 7 (scale free R2 = 0.82) was selected for screening network topology and further cluster dendrogram analysis (Figure S1B,C). Finally, a total of 13,955 genes were classified into 15 modules with similar expression trends, and ten modules were either positively (correlation ≥ 0.5, *p* < 0.05) or negatively (correlation ≤−0.5, *p* < 0.05) associated with different folate derivatives (Figure 8A). The gene expression trends of the modules related to 5-M-THF and 5-F-THF were presented, and the turquoise module showed significant expression variation from DAP 35 to mature kernel (Figure 8B,C). The gene expression trends of modules, which had no correlation with folate, are shown in the Supplementary Materials (Figure S2).

### 2.8. Bioinformatic Analysis of the Detected Co-Expressed Modules

**Figure 9:**
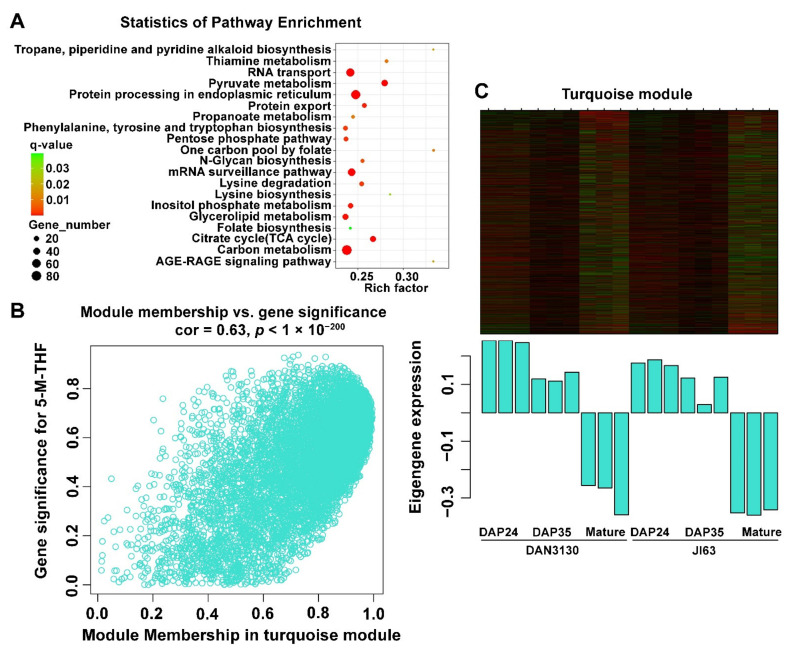
Bioinformatic analysis of the turquoise module. (**A**) KEGG enrichment of genes in the turquoise module; (**B**) The gene significance for 5-M-THF in the turquoise module (one dot represents one gene in the turquoise module); (**C**) Expression pattern of the genes and eigengenes of the turquoise module.

KEGG analysis of genes from concerned modules was performed to reveal the specific function played by each co-expressed module, and the top 20 KEGG pathways were screened out. In the turquoise module, “folate biosynthesis” and “one-carbon pool by folate” pathways were significantly enriched, especially “one-carbon pool by folate” had the highest rich factor (Figure 9A). Meanwhile, similarly to the KEGG results of development-specific and inbred-specific variation, pyruvate metabolism was identified again here with a high rich factor, which further confirms that this pathway might be related to folate accumulation (Figure 5, Figure 7 and Figure 9A). The gene significance (GS) and module membership analysis results were plotted for the turquoise module, which indicated that this module was significantly relevant with 5-M-THF (Figure 9B). Moreover, the genes involved in the turquoise module were highly down-regulated in mature kernels according to the heatmap and bar plot with module eigengene (Figure 9C). In addition, green-yellow, purple, tan, brown, red, pink, blue, magenta, and yellow modules also showed more or less relationships with different folate derivatives. The “folate biosynthesis” pathway was also identified in the KEGG enrichment of brown and blue modules (data not shown).

**Figure 10:**
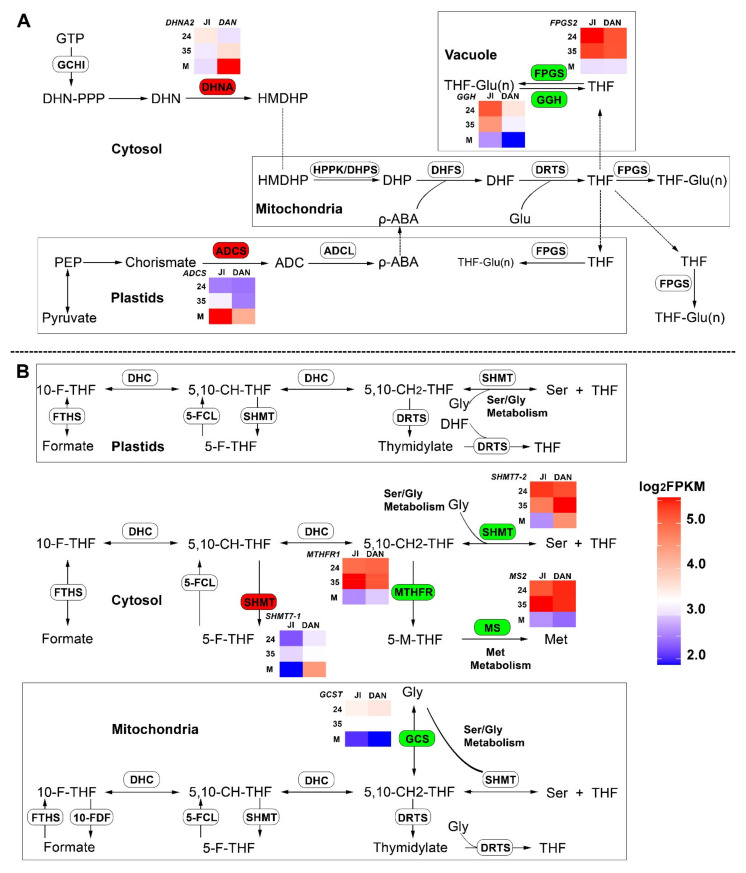
Schematic representation of the folate and one-carbon metabolic reactions in plants. (**A**) Folate biosynthesis pathway; (**B**) One-carbon metabolism pathway. Green, gene shows positive correlation with 5-M-THF; Red, gene shows positive correlation with 5-F-THF. The panel of heatmaps show the gene expression variation. JI, JI63; DAN, DAN3130; 24, DAP 24; 35, DAP35; M, mature kernel. Chemical compounds: ADC: Aminodeoxychorismate; DHF: Dihydrofolate; DHN: Dihydroneopterin; DHP: Dihydropteroate; GTP: Guanosine-5’-triphosphate; HMDHP: 6-Hydroxymethyldihydropterin; PEP: Phosphoenolpyruvate; ρ-ABA: Para-aminobenzoate; THF: Tetrahydrofolate; THF-Glu(*n*): Tetrahydrofolate polyglutamate; 5,10-CH-THF: 5,10-methynyl-THF; 5,10-CH=THF: 5,10-methylene-THF; 10-F-THF: 10-Formyl tetrahydrofolate; Ser: Serine; Gly: Glycine; Met: Methionine. Enzymes: DHC: 5,10-Methenyl-THF cyclohydrolase/5,10-methylene-THF dehydrogenase; DHFS: Dihydrofolate synthase; DHNA: Dihydroneopterin aldolase; DRTS: Dihydrofolate reductase-thymidylate synthase; FPGS: Folylpolyglutamate synthetase; FTHS: 10-Formyltetrahydrofolate synthetase; GCHI: GTP cyclohydrolase I; GCS: Glycine cleavage system; GGH: γ-Glutamyl hydrolase; HPPK/DHPS: Pyrophosphokinase/dihydropteroate synthase; MS: Methionine synthase; MTHFR: Methylenetetrahydrofolate reductase; SHMT: Serine hydroxymethyltransferase; 5-FCL: 5-formyl THF cycloligase; 10-FDF: 10-formyl THF deformylase.

After matching known folate metabolism genes with genes in each concerned module, 17 folate-related genes were found in the turquoise module, 5 were found in the blue module, and 3 were found in the brown module (Table S3). Some of the genes showed either positive (GS ≥ 0.5, p.GS ≤ 0.05) or negative (GS ≤ −0.5, p.GS ≤ 0.05) correlation with folate phenotype, which were considered as putative key genes in folate metabolism. Firstly, most genes involved in the one-carbon metabolism process showed the opposite correlation between 5-M-THF and 5-F-THF, suggesting the complexity of folate metabolism. Secondly, these genes encode protein ADCS, ADCL, GCHI, DHNA, DHFR-TS, FPGS, GGH, GCS, SHMT, FPGS, MTHFR, MS, and DHC, which were almost involved in the entire metabolism chain from folate biosynthesis to one-carbon metabolism. Genes that participate in the folate metabolic process from THF to 5-M-THF, such as *GCST*, *SHMT7-2*, *MTHFR1*, and *MS2*, as well as those that controls the polyglutamate tail length (such as *GGH* and *FPGS2*) showed the positive correlation with 5-M-THF (Figure 10, Table S3). These genes consistently showed downregulated expression levels from DAP 35 to mature kernel. For 5-F-THF, most genes had a negative correlation. In contrast, gene *ADCS* in the ρ-ABA branch, gene *DHNA2* in the pteridine branch, and gene *SHMT7-1*, which might be involved in 5-F-THF biosynthesis, were positively associated, and these three genes obviously had expressional variation between two inbred lines (Figure 10, Table S3). For the other modules, those with the absolute value of correlation ≥ 0.7, *p*-value ≤ 0.05, such as the purple, tan, red, and pink modules, were screened out for analyzing hub genes. The larger weighted value indicates the stronger proof that a gene is a trait-associated hub gene [31,32]. The networks of the top 200 edges ranked with weighted values were sorted; then, those genes with the edge numbers ≥ 15 were selected as highly connected intramodular hub genes (Table S4). Four hub genes were identified in the purple module, two of them encoding the 60S ribosomal protein, which functions in rRNA binding. In the tan module, three hub genes were identified, one belonging to the folate transporter family and another with GTPase activity. Five hub genes were found in the red module, three of which function in metal ion binding. In the pink module, six hub genes were identified: two of them related to the ribosome, one had GTPase activity, one with RNA binding activity, and one with serine/threonine kinase activity. Above all, genes related to ribosomal proteins and folate/biopterin transporters might be related to the folate concentration.

### 2.9. The Gene Expression Analysis and Validation of Folate Metabolism Genes

As mentioned above, those genes associated with 5-M-THF and 5-F-THF from module analysis results are listed in Table S5. JI63 had higher contents of 5-M-THF and 5-F-THF than DAN3130 on DAP 24, while it was the opposite on mature kernels. For immature DAP 24, 5-M-THF was the major folate derivatives that accounts for 85%–90% of total folate, *GGH* and *FPGS2*, which showed the highest positive correlation (GS = 0.62) with 5-M-THF, which had significantly higher gene expression in JI63 than DAN3130. For mature kernels, the proportion of 5-F-THF had sharply increased from 4%–8% to 34%–36%; *DHNA2* and *SHMT7-1*, which both showed a highly positive correlation with 5-F-THF (GS = 0.63 and 0.64, respectively), had higher FPKM value in DAN3130 than JI63.

The RNA-seq data were verified by qPCR using eight representative DEGs from the folate metabolism pathway. The expression level profiles of these eight genes were similar as determined by RNA-seq and qPCR, and the two methods showed high consistency (Figure S3).

## 3. Discussion

The development of maize kernels includes early development, differentiation, periods of mitosis and endoreduplication, accumulation of storage compounds, and maturation [33]. It spans the early stage (DAP 1 to 11), filling stage (DAP 12 to 39), and desiccation stage (DAP 40 to 70). During the filling stage, sucrose and amino acids are converted to starch and storage proteins. The genes related to amino acid biosynthesis accumulate to their optimal level from DAP 10 [34], starch accumulation peaks at DAP 15 and then remains steady [35,36], and oil concentration reaches its peak level on DAP 30 [37]. At the end of seed filling, seed maturation starts with the progressive loss of water [38]. The pyruvate metabolism and glutamate metabolism were identified from the development-specific folate variation in kernel filling stage (DAP 24 to DAP 35) and desiccation stage (DAP 35 to mature kernel) in DAN3130 and JI63 (Figure 4A). Pyruvate is the end-product of glycolysis and a major substrate for oxidative metabolism, and it is converted into phosphoenolpyruvate, an upstream precursor for chorismate biosynthesis [39,40]. In Arabidopsis (*Arabidopsis thaliana*) and potato, pyruvate might be degraded to formate by pyruvate formate-lyase to provide the source of one-carbon source for folate regulation [41]. Glutamate is one of the most abundant amino acids that play an essential role in folate biosynthesis as glutamate tail, and the number of glutamate tails has been known as a key factor of folate stability [42]. The Venn diagram of common genes, which are found in alanine/aspartate/glutamate metabolism from development-variation analysis and WGCNA analysis, showed that three of five common genes are involved in glutamate metabolism (Figure S4). The transcription level of these three genes increased from DAP 24 to DAP 35. We hypothesized that the more active transcription of these genes causes a reduction in the glutamate level, resulting in decreased folate from DAP 24 to DAP 35.

The accumulation pattern of folate derivatives differed from DAP 35 to mature kernels in the two lines, suggesting that some specific pathways could affect 5-M-THF and 5-F-THF separately during this stage. As shown in Figure 4B, acyltransferase activity term and methyltransferase activity term were found in DAN3130 and JI63, respectively, during DAP 35 to mature kernel, which refers to the 5-F-THF increase in DAN3130 and 5-M-THF decrease in JI63. A total of 55 genes were identified in terms of acyltransferase activity. Among them, 11 genes belonged to the Acyl-CoA *N*-acetyltransferase (NAT) superfamily protein/GNAT-transcription factor family (data not shown). In plants, folates are degraded into ρ-aminobenzoylglutamate (ρ-ABAGlu) in vivo, ρ-ABAGlu and its polyglutamates are hydrolyzed into glutamate and ρ-ABA [43]. NAT type 1 (NAT1) in humans and Nat2 in mice reportedly use the ρ-ABAGlu as the substrate [44]. Increasing the ability to acetylate ρ-ABAGlu enhances folate metabolism and demonstrates that NAT regulates folate levels by acetylating ρ-ABAGlu in animals [45], while whether NAT in plants is related to ρ-ABAGlu remains unknown. Aminoacyl-tRNA, which is formylated to form THF to participate in one-carbon metabolism [46], also is acetylated by GNAT toxins in Arabidopsis [47]. Here, genes belonging to the GNAT-transcription factor family were significantly enriched, while more evidence is required to validate if GNAT contributes to folate accumulation in maize. There are a total of 28 genes in terms of *N*-methyltransferase activity, among which 17 genes encoded histone-lysine *N*-methyltransferase (data not shown). In plants, histone-lysine methyltransferases catalyze the transfer of the methyl group from S-adenosylmethionine (SAM), which is generated from folate and methionine cycles [48]. In Arabidopsis, the loss function of *ATMS1* causes an accumulation of Hcy and SAH, leading to reduced SAM accessibility to histone methyltransferases [49]. In animals, the loss of histone H3 lysine 4 trimethylation at promoters has been linked to decreases in Transcription Factor II D (TFIID) [50]. Taken together, the results demonstrate that the variation in folate accumulation during the late stage of maize kernel development could be mainly controlled by circumjacent pathways of folate, especially pyruvate metabolism and glutamate metabolism. Genes belonging to the GNAT-transcription factor family and histone-lysine *N*-methyltransferase family might also be associated with folate metabolism.

The folate biosynthesis pathway and one-carbon pool of folate were specially found in the KEGG results of the inbred-specific folate variation. WGCNA analysis showed that those genes belonging to folate biosynthesis (pteridine branch and ρ-ABA branch) and the one-carbon metabolism process had highly positive/negative correlations to folate phenotype. In folate biosynthesis, *ADCS* and *DHNA2* showed a positive correlation with 5-F-THF and THF, confirming the plant folate biofortification strategy by engineering of pteridine and ρ-ABA branches [2,4,51]. For example, *ADCS* has been applied in Arabidopsis, rice, potato, tomato, maize, and Mexican common bean (*Phaseolus vulgaris*) to increase folate accumulation [51,52,53,54,55,56,57]. Overexpression of the heterologous *Escherichia. coli DHNA* (*EcDHNA*) in tobacco (*Nicotiana tabacum*) enhances the folate accumulation [58]. *DRTS-3* and *DRTS-4* showed negative correlations with 5-F-THF and THF, which is consistent with the findings in Arabidopsis: *THY3* (*DRTS-3*) inhibits the expression of its two homolog genes and fine-tunes the folate concentration during development. Overexpression of the *THY3* results in a reduced folate concentration [17]. Furthermore, genes involved in the pteridine and ρ-ABA branches, such as *ADCS*, *GCHI-1* and *DHNA1*, were negatively correlated with 5-M-THF, suggesting that the negative feedback control might exist between 5-M-THF and folate biosynthesis genes. The ADCS activity is inhibited by DHF in vitro, but whether this is also the case in vivo is unclear [9], and the expression of *GCHI* and *ADCS* varies significantly in tomato fruit [23,59]. Notably, genes involved in the interconversion of monoglutamate-folate and polyglutamate-folate, such as *FPGS2* and *GGH*, had a significant correlation with folate content. In Arabidopsis and tomato, FPGS and GGH play an essential function in folate homeostasis. Indeed, knock-out of the mitochondrial *FPGS* or overexpression of the *GGH* could reduce 40–45% of the total folate in Arabidopsis leaves, and the reduction of Arabidopsis GGH enzyme activity resulted in 30% increase of total folate [60,61]. The transcript level of *GGH1*, which removes the polyglutamyl tail from folates, is an indicator of folate content in potato tubers [27]. The *GGH* and *FPGS2*, which are related to glutamate utilization, identified in WGCNA analysis, were consistent with the glutamate pathway identified in the KEGG results.

In addition, the expression level of these genes that participate in one-carbon metabolism are classed into serine/THF/5-M-THF cycle (*SHMT*, *GCS*, *MTHFR,* and *MS*) that show a positive correlation with folate accumulation. SHMT is involved in glycine oxidation, photorespiration, and one-carbon metabolism that produces 5,10-CH=THF from THF. In *Saccharomyces cerevisiae*, the stimulation of *SHMT2* expression leads to folate accumulation [62]. The glycine cleavage system catalyzes the THF-dependent catabolism of glycine into 5,10-CH=THF to join the folate cycle, and the P protein (GCSP) binds the alpha-amino group of glycine via its pyridoxal-5’-phosphate cofactor [63]. A novel rice tetrahydrofolate cycle mutant *hpa1* in which HPA1 (encoding a tetrahydrofolate deformylase) is mutated shows the variation in THF, 5-F-THF, and 10-F-THF as well as the downregulation of *SHMT* and *GCS* [64]. *MTHFR* and *MS* directly participate in 5-M-THF metabolism. Notably, these two genes were identified in this study (Figure 10, Table S3), reflecting the connection between folate accumulation and conversion of homocysteine into methionine. The importance of folate polyglutamylation in methionine biosynthesis has been confirmed. The metabolic profiling of folate-deficient cells shows that the SAM and methionine pools decline during the initial period of folate starvation in Arabidopsis, indicating that methionine synthesis is mainly associated with the perturbation of the folates pool [65]. *SHMT* and *GCS* also participate in the interconversion of serine and glycine, which is consistent with the identification of the Ser/Gly metabolism pathway in the KEGG analysis (Figure 5, Figure 10, Table S3). Moreover, one of the genes encoding the folate/biopterin transporter (FBT) protein was screened out as a hub gene from modules analysis (Table S4). In *Leishmania*, FBT family members are shown experimentally to transport folate, but their functions in maize are unknown [66].

The disturbance in metabolisms of either the pyruvate or amino acids may result from the varied folate metabolism at transcript levels, which is in accordance with the previous studies. For example, pyruvate orthophosphate dikinase (PPDK) is an important enzyme participating in pyruvate metabolism that catalyzes phosphoenolpyruvate regeneration. The dysfunction of *PPDK* in maize resulted in a dramatical increase in expression of cytosolic glutamine synthetase (*GS1*) and asparagine synthetase (*ASN1*), and eight folate-related genes showed differential expression patterns in leaves compared with wild type (Table S6) [67]. The *GS1*, *ASN,* and seven of eight folate-related genes were also identified in this study, and they showed strong correlation (either positive or negative) with folate derivatives, indicating the connection between pyruvate and folate metabolisms (Figure S4, Tables S3 and S6). Similar connections were also identified between amino acid and folate metabolisms. For instance, *O2* and *O16* encode the transcription factors of the basic leucine zipper family, and they have been used to improve the amino acid quality of maize grains [68]. The kernels of double recessive mutant *o2o2o16o16* contained increased Gly as well as reduced Glu and Met. Coincidently, a total of 24 folate-related genes showed significant variation of expression between mutant and wild type, and 21 of them were also found in our WGCNA module analysis, including the key folate genes *ADCS*, *DHNA2*, *GGH*, *SHMT7-1*, *SHMT7-2,* and *GCST* (Tables S3 and S7) [68]. Similarly, studies in tomato and millet (*Setaria italica*) also demonstrated that folate accumulation in different cultivars was closely related to the genes involved in pteridine branch, ρ-ABA branch, folate degradation, and serine/THF/5-M-THF cycle, such as *SiDHNA2*, *SiGGH*, *SiSHMT2*, *SiSHMT3*, *SiMTHFR*, *SiAMT* (known as *GCST*), *SlDHNA*, and *SlADCS* [25,69].

In conclusion, the comparative transcriptome analysis in this study provides the complexity of folate metabolism and creates awareness of the interaction among metabolism mentation above during the futural maize breeding.

## 4. Materials and Methods

### 4.1. Plant Material and Folate Measurement

DAN3130 and JI63 inbred lines are originated from China, belonging to the NSS subpopulation, JI63 with pedigree being (127-32 × Tie84) × (Wei24 × Wei20) and DAN3130 with pedigree being American hybrid P78599 [70]. They were grown at Langfang, Hebei province, in the summer of 2018. Fresh kernel samples were collected 24 and 35 days after pollination and then frozen in liquid nitrogen immediately. Mature kernel samples were harvested after all the plants turned yellow. Three biological replicates of each stage were harvested. The folates exaction and measurement were repeated three times in each replicate. The methods of folate extraction and measurement were the same as in the previous study [53].

### 4.2. RNA Extraction and Library Preparation

The total RNA of DAP 24, DAP 35, and mature kernels were extracted using a BAIXU Maize Kernel Total RNA Extraction Kit (Beijing, China). To eliminate any residual genomic DNA, the total RNA was treated with RNase-free DNase I (New England Biolabs, Ipswich, MA, USA), and the concentration of RNA was determined using a Nanodrop-2000. The total RNA was used to synthesize first-strand complementary DNA (cDNA) using the RevertAid First Strand cDNA Synthesis kit (Fermentas, Waltham, MA, USA).

### 4.3. Raw Read Filtering and Assembly

RNA-Seq library preparation and sequencing were carried out by BEIJING BAIXU BIOTECH CO., LTD, Beijing, China. All libraries were sequenced using the Sanger method by Illumina 1.9 Platform. The adapters, low-quality reads, and the reads containing poly-N were removed to obtain clean reads. Then, the clean reads were mapped to the *Zea mays* B73 genome by using Hisat software (v2.1.0, http://daehwankimlab.github.io/hisat2/, accessed on 10 November 2019) with setting non-strand-specific and other default parameters. Afterwards, the clean reads were assembled using Samtools software (http://www.htslib.org/, accessed on 14 November 2019), Cufflink software (v2.2.1, http://cole-trapnell-lab.github.io/cufflinks/, accessed on 14 November 2019) and Cuffcompare software (v2.2.1, http://cole-trapnell-lab.github.io/cufflinks/cuffcompare/, accessed on 14 November 2019).

### 4.4. Gene Annotation and DEG Analysis

The sequences were aligned to *Zea mays* B73 genome v4.40 (data download from: ftp://ftp.ensemblgenomes.org/pub/plants/release-40/gtf/zea_mays/Zea_mays.AGPv4.40.chr.gtf.gz Accessed date: 10 February 2020). Genes were annotated based on the maize B73 genome v4.40 annotation in Ensemblgenomes (data download from: ftp://ftp.ensemblgenomes.org/pub/plants/release-40/gff3/zea_mays/Zea_mays.AGPv4.40.chr.gff3.gz Accessed date: 10 February 2020). The rest of the unmatched transcripts were annotated using Trinotate software (v3.2.0, https://rnabio.org/module-07-trinotate/0007/02/01/Trinotate/ Accessed date: 29 March 2020, including TransDecoder v5.5.0, HMMER v3.1b2, Blast v2.9.0, SignalP v4.1, TMHMM v2.0c, and RNAmmer v1.2), Uniprot database (https://www.uniprot.org/ Accessed date: 29 March 2020), and NCBI RefSeq non-redundant proteins databases (data download from ftp://ftp.ncbi.nlm.nih.gov/blast/db/FASTA/nr.gz Accessed date: 29 March 2020). Htseq-count software (v0.11.2) was used for counting reads, and DESeq-2 software was used for standardization and DEG analysis. The FPKM (Fragments Per Kilobase of exon model per Million mapped reads) of each gene was calculated according to its length and the mapped read numbers. |Log2FC (Fold Change)| ≥ 1 and *p*-value <0.05 were used as the threshold to screen DEGs.

### 4.5. Bioinformatics Analysis

GO (GO, http://www.geneontology.org/ Accessed date: 21 April 2020) and functional enrichment analysis were conducted on all DEGs using Agrigod(v2) software (http://systemsbiology.cau.edu.cn/agriGOv2/index.php Accessed date: 21 April 2020). Then, all DEGs were mapped to a pathway in the KEGG database (http://www.genome.jp/kegg/pathway.html Accessed date: 21 April 2020) using KOBAS 3.0 software (http://kobas.cbi.pku.edu.cn/kobas3 Accessed date: 21 April 2020). The *p*-value was set as ≤ 0.05 as the threshold to judge the significance of the GO and KEGG pathway enrichment analyses. Gene co-expression networks were performed using the WGCNA package in the R software. The steps were followed as below: https://horvath.genetics.ucla.edu/html/CoexpressionNetwork/Rpackages/WGCNA/Tutorials/ Accessed date: 5 May 2021 [71]. A total of 13955 genes were filtered by mean expression (mean normalized counts > 10) and then log-transformed using log2(x + 1) for WGCNA analysis.

### 4.6. Real-Time Quantitative PCR Analysis

qPCR was performed in ABI QuantStudio 6 real-time PCR system using TransStart Top Green qPCR SuperMix (TransGen Biotech, Beijing, China). The cDNAs were made from three samples, and all reactions were performed in quadruplicate. PCR conditions were as follows: 95 °C for 30 s, 40 cycles of 95 °C for 5 s, and 60 °C for 34 s. The primers of selected genes for qPCR are listed in Table S8. *GAPDH* (*Zm00001d049641* encodes the glyceraldehyde-3-phosphate dehydrogenase) was used as the reference gene to normalize the target gene expression, which was calculated using the relative quantization method (2^−ΔΔ^CT).

## 5. Conclusions

Two maize inbred lines, DAN3130 and JI63, showed significant folate variation and different folate accumulation patterns during the late stage of kernel formation, indicating the complexity of mechanism of folate metabolism. The results demonstrated that the folate development-specific variation from DAP 24 to mature kernels might be regulated by the circumjacent pathways of folate biosynthesis such as pyruvate metabolism, glutamate metabolism, and glycine/serine metabolism, as well as by genes belonging to the GNAT-transcription factor family and histone-lysine *N*-methyltransferase family. Genes involved in the two branches of folate biosynthesis, serine/THF/5-M-THF cycle and conversion of THF monoglutamate into THF polyglutamate, could affect the folate levels between different lines. The results provide insight into the mechanism of folate accumulation and will help the futural breeding work of folate biofortification.

## Data Availability

The raw data were uploaded and deposited in the National Center for Biotechnology Information (NCBI) Gene Expression Omnibus (GEO) under the accession number: GSE195815.

## Supplementary Materials

The following are available online at https://www.mdpi.com/article/10.3390/ijms23031708/s1.
