# Supplementary material for: Comparative Transcriptome Analysis Reveals Mechanisms of Folate Accumulation in Maize Grains
Source: Int J Mol Sci. 2022 Feb 1;23(3):1708. doi: 10.3390/ijms23031708 (PMC8836222; doi:10.3390/ijms23031708)
Supplement: Supplementary file 1 [file ijms-23-01708-s001.zip › ijms-1537453-supplementary.pdf]

International Journal of *Molecular Sciences*

MDPI

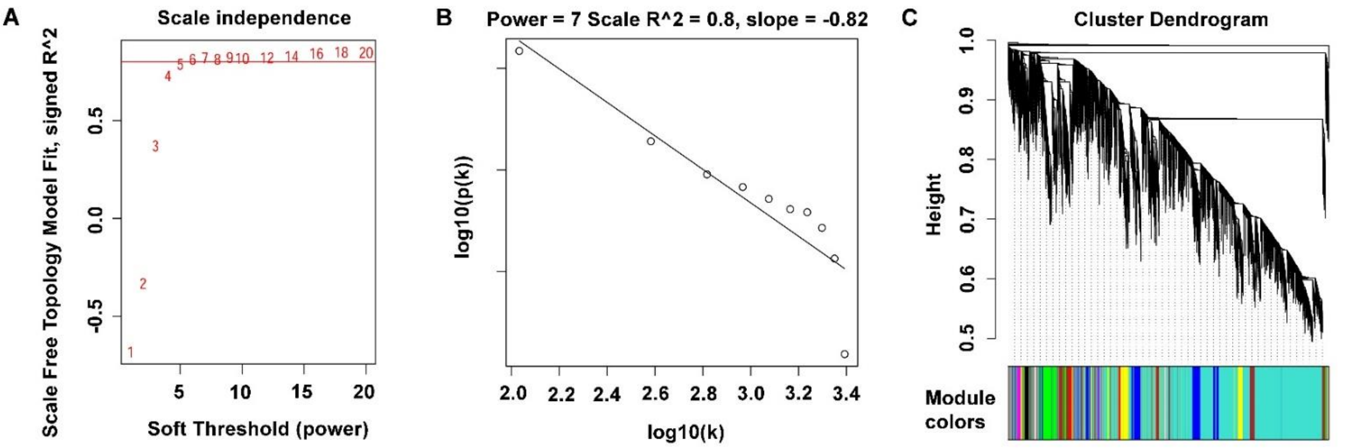

Figure S1. WGCNA analysis. A. Soft-thresholding power analysis; B. Scale free topology when soft-thresholding power $\beta = 7$; C. Clustering dendrogram of genes, with dissimilarity based on the topological overlap, together with assigned module colors.

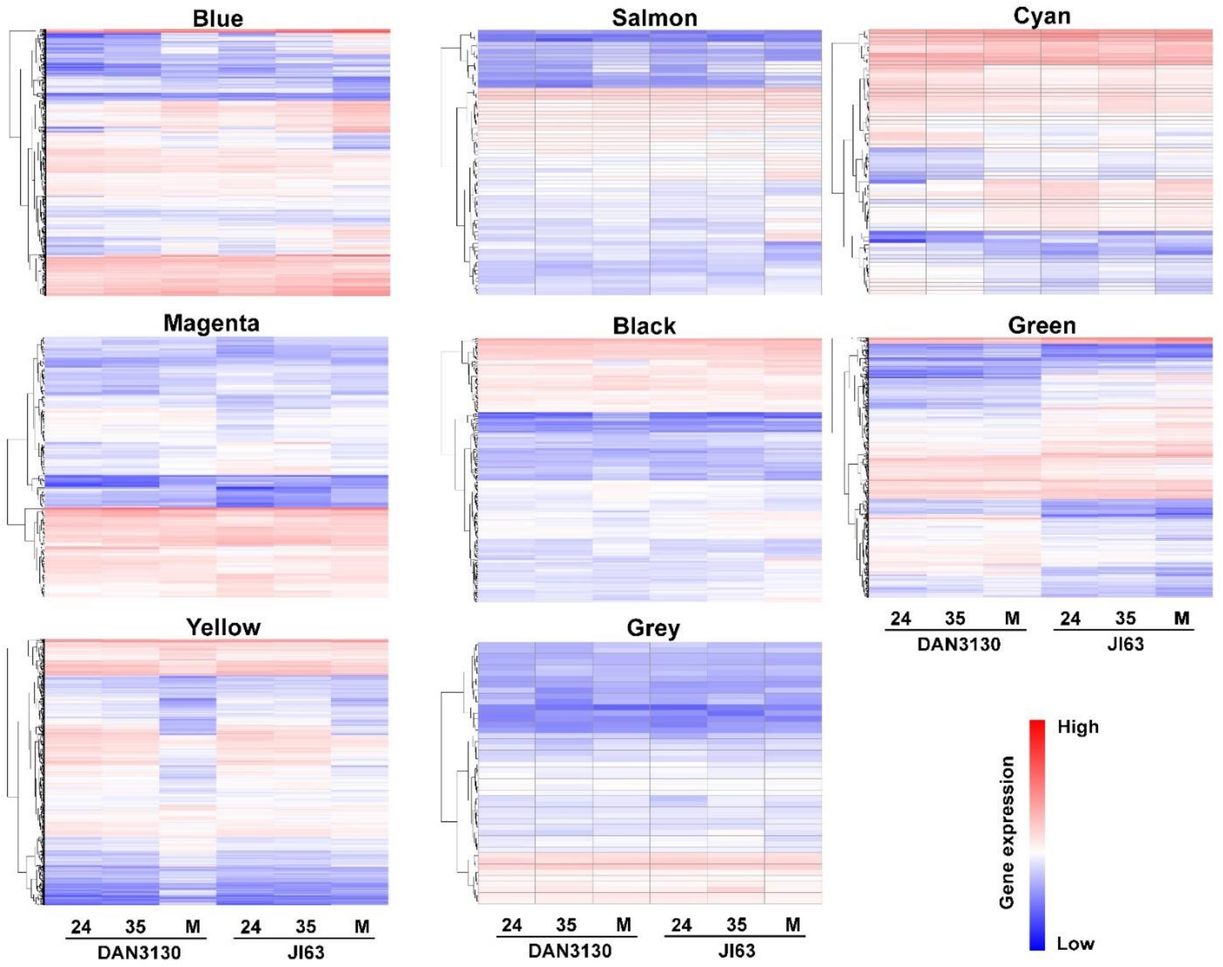

Figure S2. The gene expression heatmap of modules, which has no correlation with folate, from WGCNA results.

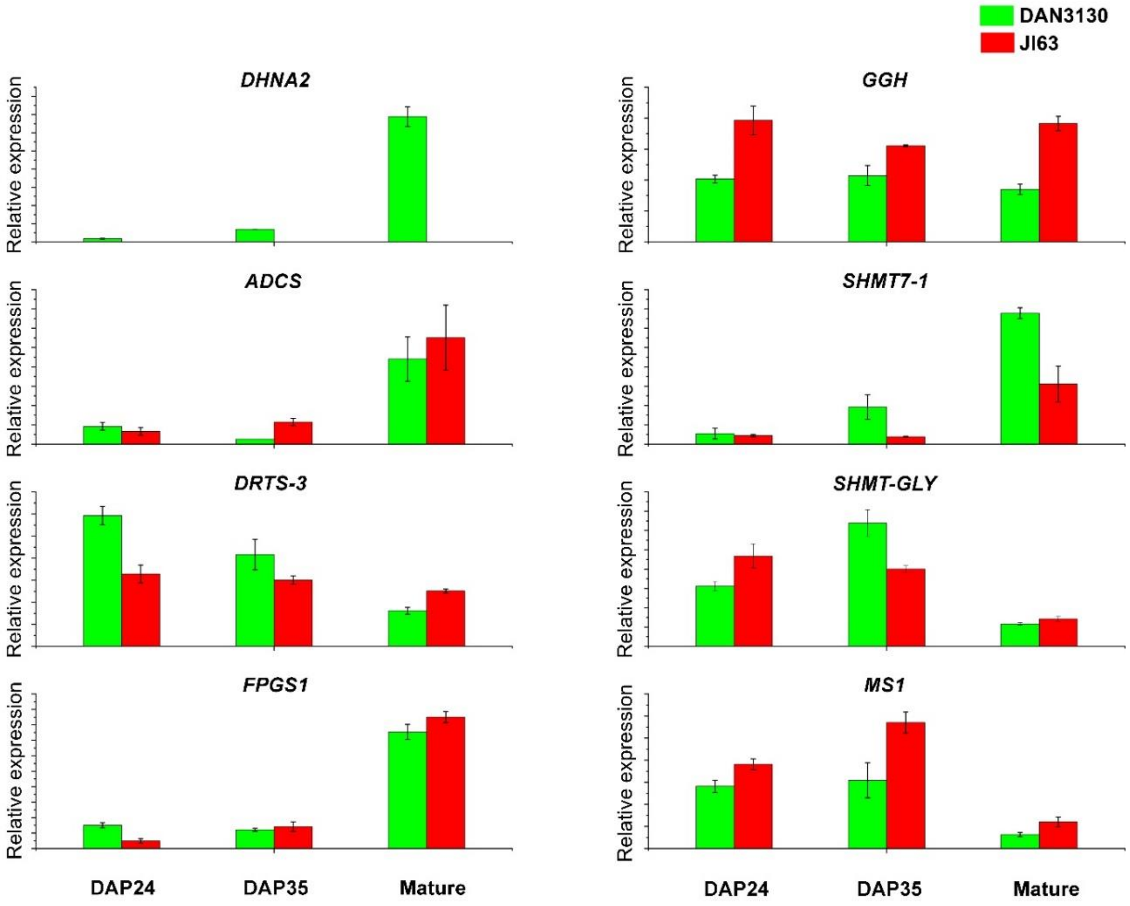

Figure S3. qPCR verification of representative DEGs from the folate metabolism pathway.

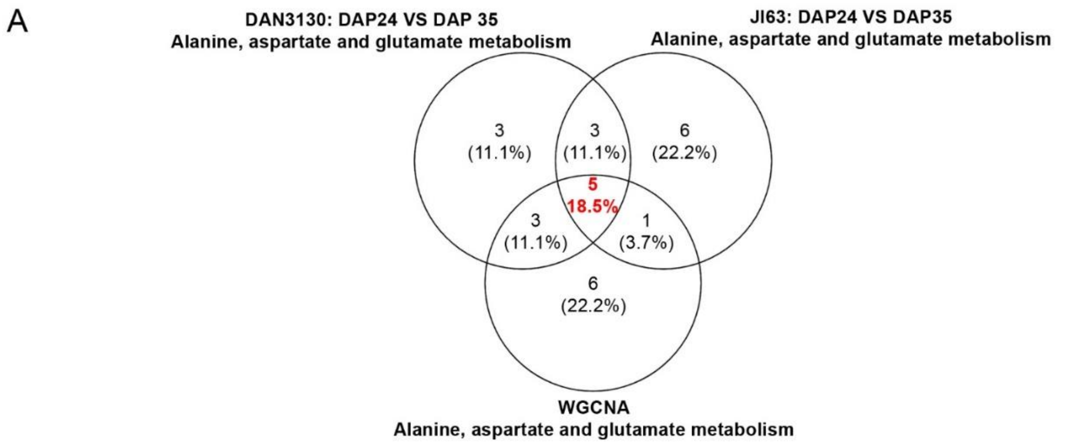

B FPKM value of five common genes in alanine, aspartate and glutamate metabolism pathway

Gene	Description	DAN3130			JI63		
		DAP 24	DAP 35	Matural kernel	DAP 24	DAP 35	Matural kernel
Zm00001d044608	Asparagine synthetase 2	33.88±7.37	235.40±36.28	453.36±140.30	61.68±9.85	217.41±37.32	373.29±14.96
Zm00001d028750	Asparagine synthetase 3	3.47±1.51	8.30±9.49	83.87±19.03	4.83±1.46	19.34±2.19	154.75±22.47
Zm00001d031749	Glutamate decarboxylase	28.09±0.49	115.13±11.43	276.49±85.74	56.77±3.52	235.72±44.64	494.40±41.46
Zm00001d051804	Glutamine synthetase5	4.43±0.24	11.18±2.35	26.75±4.31	7.72±1.27	17.93±5.61	29.09±8.15
Zm00001d010197	Glutamine 5-phosphoribosylpyrophosphate amidotransferase	4.52±0.90	11.26±0.54	115.59±45.78	4.75±1.63	14.38±6.91	120.32±4.62

Figure S4. The Venn diagram of common genes in glutamate metabolism. A. The Venn diagram of common genes in glutamate metabolism in alanine/aspartate/glutamate metabolism from development-variation analysis and WGCNA analysis. B. The FPKM value of five common genes in alanine/aspartate/glutamate metabolism.

Table S1. Folate profiling of maize kernel during late stages of development.

Folate Derivatives	DAP 24 (nmol/g FW)		DAP 35 (nmol/g FW)		Mature Kernel (nmol/g DW)	
	DAN3130	JI63	DAN3130	JI63	DAN3130	JI63
5-M-THF	0.89 ± 0.08	1.35±0.15	0.48±0.03	0.72±0.08	0.54±0.02	0.22±0.02
5-F-THF	0.04±0.01	0.12±0.02	0.03±0.01	0.16±0.01	0.34±0.02	0.16±0.001
THF	0.03±0.005	0.04±0.01	0.02±0.002	0.04±0.007	0.07±0.004	0.03±0.004
5,10-CH=THF	0.03±0.003	0.06±0.01	0.03±0.005	0.08±0.001	0.04±0.005	0.02±0.005

Table S2. Overview of transcriptome sequencing data.

Sample ID	Rep	Clean Reads	Q20 Bases	Q30 Bases	GC Content	Mapping Reads	Mapped Reads with Unique Loci	Mapping Ratio
DAN3130 DAP 24	1	74,987,086	95.05%	88.88%	51.51%	64,521,241	54,258,276	86.04%
	2	174,991,970	95.04%	88.88%	51.24%	149,238,661	123,619,968	85.28%
	3	48,358,824	93.64%	87.34%	45.67%	36,727,718	30,061,383	75.95%
DAN3130 DAP 35	1	46,343,382	92.68%	86.46%	46.32%	31,747,792	26,817,158	68.51%
	2	44,861,502	93.64%	87.37%	47.87%	33,448,957	27,666,652	74.56%
	3	87,136,232	94.91%	89.10%	51.86%	71,739,096	60,279,115	82.33%
DAN3130 Mature kernels	1	43,319,674	93.62%	87.93%	48.30%	31,086,099	17,759,180	71.76%
	2	76,918,438	92.27%	85.37%	46.30%	52,517,640	17,921,475	68.28%
	3	117,938,336	96.78%	92.45%	59.38%	94,704,697	56,161,081	80.30%
JI63 DAP 24	1	90,251,840	94.90%	89.11%	50.23%	72,548,253	63,163,421	80.38%
	2	46,572,962	93.29%	87.92%	43.57%	31,705,696	26,162,933	68.08%
	3	153,045,578	93.29%	86.88%	46.12%	109,131,672	91,159,663	71.31%
JI63 DAP 35	1	236,947,674	94.58%	88.46%	52.47%	191,990,396	172,220,593	81.03%
	2	71,881,146	93.60%	86.93%	58.45%	51,008,354	47,576,576	70.96%

	3	175,373,372	94.87%	89.08%	50.63%	140,232,312	126,527,577	79.96%
JI63 Mature kernel	1	73,863,982	96.16%	91.10%	60.54%	57,660,477	30,866,985	78.06%
	2	64,518,056	96.64%	92.33%	58.13%	50,827,949	30,464,526	78.78%
	3	70,535,482	95.86%	90.55%	60.11%	53,851,285	29,096,977	76.35%

Table S3. Folate metabolism genes in concerned modules.

Gene	ID	M.C.	S.L.	MM	p.MM	5-M-THF		5-F-THF		THF		5,10-CH=THF		Total	
						GS	p.GS	GS	p.GS	GS	p.GS	GS	p.GS	GS	p.GS
ADCS	Zm00001d036484	turquoise	C	-0.929	2.46E-08	-0.676	2.09E-03	0.491	3.87E-02	0.335	1.74E-01	-0.324	1.89E-01	-0.375	1.25E-01
ADCL1	Zm00001d029231	green	O	-0.820	3.07E-05	0.158	5.31E-01	-0.415	8.68E-02	-0.506	3.21E-02	0.307	2.16E-01	-0.070	7.82E-01
ADCL2	Zm00001d039264	turquoise	O	-0.415	8.71E-02	-0.474	4.69E-02	-0.272	2.75E-01	-0.272	2.74E-01	-0.550	1.80E-02	-0.608	7.45E-03
GCHI-1	Zm00001d001959	blue	O	0.838	1.40E-05	-0.771	1.82E-04	0.352	1.52E-01	0.102	6.87E-01	-0.044	8.62E-01	-0.528	2.44E-02
GCHI-2	Zm00001d026531	blue	O	0.777	1.51E-04	-0.235	3.48E-01	-0.147	5.61E-01	-0.185	4.63E-01	0.138	5.86E-01	-0.290	2.44E-01
DHNA1	Zm00001d031979	turquoise	O	-0.865	3.53E-06	-0.608	7.40E-03	0.252	3.13E-01	0.227	3.65E-01	-0.458	5.57E-02	-0.443	6.57E-02
DHNA2	Zm00001d031995	turquoise	O	-0.604	7.95E-03	-0.242	3.33E-01	0.627	5.35E-03	0.667	2.48E-03	-0.102	6.88E-01	0.116	6.48E-01
HPPK/DHPS	Zm00001d021338	turquoise	O/M	0.823	2.78E-05	0.399	1.01E-01	-0.333	1.77E-01	-0.337	1.72E-01	0.363	1.39E-01	0.202	4.23E-01
DHFS1	Zm00001d023817	blue	M	-0.166	5.10E-01	0.078	7.57E-01	0.035	8.90E-01	0.222	3.76E-01	-0.270	2.78E-01	0.086	7.33E-01
DHFS2	Zm00001d041625	magenta	M	-0.555	1.68E-02	0.551	1.77E-02	-0.233	3.53E-01	0.033	8.97E-01	0.222	3.76E-01	0.401	9.87E-02
DRTS3	Zm00001d007318	turquoise	O	0.848	8.69E-06	0.406	9.42E-02	-0.516	2.84E-02	-0.463	5.29E-02	0.303	2.21E-01	0.109	6.67E-01
DRTS4	Zm00001d049188	turquoise	M	0.668	2.44E-03	0.436	7.03E-02	-0.698	1.26E-03	-0.630	5.06E-03	0.187	4.57E-01	0.032	8.99E-01
FPGS1	Zm00001d032529	turquoise	C/O	-0.743	4.14E-04	-0.745	3.92E-04	0.436	7.04E-02	0.308	2.14E-01	-0.357	1.46E-01	-0.468	5.02E-02
FPGS2	Zm00001d048514	brown	O	-0.847	9.25E-06	0.618	6.30E-03	-0.633	4.81E-03	-0.563	1.49E-02	0.469	4.98E-02	0.247	3.22E-01
GGH	Zm00001d010744	brown	SP	-0.927	3.18E-08	0.554	1.71E-02	-0.412	8.91E-02	-0.419	8.39E-02	0.551	1.78E-02	0.309	2.12E-01
GCSP	Zm00001d023437	turquoise	O	0.828	2.24E-05	0.437	6.96E-02	-0.841	1.21E-05	-0.774	1.62E-04	0.265	2.88E-01	-0.041	8.72E-01
GCST	Zm00001d002258	turquoise	M	0.787	1.05E-04	0.520	2.71E-02	-0.823	2.73E-05	-0.696	1.34E-03	0.216	3.90E-01	0.045	8.59E-01
GCSH2	Zm00001d015378	turquoise	M	0.827	2.28E-05	0.411	8.98E-02	-0.705	1.09E-03	-0.698	1.29E-03	0.285	2.51E-01	0.008	9.75E-01
SHMT-GLY1	Zm00001d049234	turquoise	C	0.757	2.76E-04	0.378	1.22E-01	-0.858	5.41E-06	-0.821	2.95E-05	0.214	3.94E-01	-0.108	6.71E-01
SHMT7-1	Zm00001d042661	brown	O	0.818	3.30E-05	-0.526	2.49E-02	0.639	4.34E-03	0.569	1.37E-02	-0.241	3.35E-01	-0.150	5.54E-01
SHMT7-2	Zm00001d012247	blue	O	-0.847	9.15E-06	0.541	2.04E-02	-0.057	8.24E-01	0.059	8.15E-01	0.306	2.17E-01	0.485	4.15E-02
MTHFR1	Zm00001d034602	turquoise	O	0.931	2.05E-08	0.540	2.07E-02	-0.537	2.17E-02	-0.483	4.25E-02	0.534	2.25E-02	0.231	3.56E-01
MS1	Zm00001d031129	turquoise	O	0.846	9.90E-06	0.384	1.16E-01	-0.673	2.19E-03	-0.642	4.09E-03	0.363	1.39E-01	0.005	9.85E-01
MS2	Zm00001d013644	turquoise	O	0.934	1.44E-08	0.500	3.47E-02	-0.705	1.07E-03	-0.665	2.61E-03	0.368	1.33E-01	0.094	7.11E-01
DHC1	Zm00001d005614	turquoise	O/M	-0.865	3.51E-06	-0.836	1.53E-05	0.276	2.68E-01	0.059	8.16E-01	-0.464	5.26E-02	-0.648	3.63E-03
DHC2	Zm00001d010867	blue	O	0.939	8.08E-09	-0.547	1.88E-02	0.198	4.30E-01	0.076	7.64E-01	-0.022	9.30E-01	-0.399	1.01E-01
DHC3	Zm00001d053900	turquoise	C	0.589	1.00E-02	0.024	9.24E-01	-0.773	1.69E-04	-0.778	1.42E-04	-0.066	7.95E-01	-0.401	9.93E-02

5-FCL-1	Zm00001d006676	greenyellow	C	-0.710	9.70E-04	-0.188	4.54E-01	0.145	5.65E-01	0.032	9.00E-01	0.289	2.45E-01	-0.085	7.39E-01
5-FCL-2	Zm00001d023534	brown	C	0.645	3.82E-03	-0.161	5.22E-01	0.245	3.28E-01	0.301	2.25E-01	-0.478	4.50E-02	-0.035	8.89E-01
FTHS	Zm00001d020669	cyan	O	0.793	8.50E-05	-0.036	8.86E-01	-0.278	2.65E-01	-0.195	4.38E-01	-0.028	9.13E-01	-0.181	4.71E-01

MM: module membership; p.MM: p-value of module membership; GS: gene significance; p.GS p-value of gene significance. M.C.: module color; S.L.: subcellular localization by TargetP 2.0; M: mitochondrial transit peptide, C: chloroplast transit peptide, SP: signal peptide, O: other.

Table S4. Hub genes in purple, tan, red and pink module.

Module	Gene ID	Annotation	Function
Purple	Zm00001d049830	Nascent polypeptide-associated complex subunit alpha-like protein 3	Unfolded protein binding
	Zm00001d037376	Early nodulin-like protein 7	Electron transfer activity
	Zm00001d035201	60S acidic ribosomal protein P0	Large ribosomal subunit / rRNA binding
	Zm00001d003127	60S ribosomal protein L23a-1	rRNA binding
Tan	Zm00001d033850	Tubulin alpha chain	GTPase activity
	Zm00001d048584	Thioesterase family protein, mRNA	Acyl-CoA hydrolase activity
	Zm00001d019510	Folate/biopterin transporter family protein	Transport
Red	Zm00001d052905	Squamosa promoter-binding protein-like (SBP domain) transcription factor	DNA binding / metal ion binding
	Zm00001d038776	Putative mitochondrial-processing peptidase subunit alpha-2	Metal ion binding
	Zm00001d028307	Putative inactive poly [ADP-ribose] polymerase SRO1	NAD+ ADP-ribosyltransferase activity
	Zm00001d007009	DNAJ heat shock N-terminal domain-containing protein	Stress response
	Zm00001d042475	Putative thimet oligopeptidase	Metal ion binding
Pink	Zm00001d038272	Beta-glucosidase	beta-glucosidase activity
	Zm00001d022611	La-related protein 1C	RNA binding
	Zm00001d033726	Putative serine/threonine protein kinase IREH1	Protein serine/threonine kinase activity
	Zm00001d037972	60S ribosomal protein L29	Structural constituent of ribosome
	Zm00001d039211	Notchless-like protein isoform 1	Ribosomal large subunit assembly
	Zm00001d046996	Tubulin beta chain	GTPase activity

Table S5. The FPKM value of folate genes identified by WGCNA analysis.

	DAP 24		DAP 35		Mature Kernel	
**Gene**	**DAN3130**	**JI63**	**DAN3130**	**JI63**	**DAN3130**	**JI63**
ADCS	5.39±0.69	5.61±0.78	5.56±1.05	8.32±2.66	12.13±3.29	18.64±4.79
ADCL2	26.92±0.73*	16.05±4.01*	25.30±2.25	23.57±12.32	17.39±9.70	21.05±4.27
GCH-1	19.05±0.39	24.20±3.71	46.71±9.19	60.07±4.60	41.40±13.85	44.18±3.58
DHNA1	14.50±1.32	13.38±3.95	18.26±0.72	15.47±3.74	26.08±8.83	19.70±1.71
DHNA2	6.09±2.32	12.09±7.61	13.99±2.30**	6.87±1.07**	118.57±52.95*	5.72±0.56*
DRTS3	24.82±1.97*	17.07±2.59*	18.90±2.00	21.56±5.99	7.65±2.12	5.78±1.94
DRTS4	22.13±2.98	21.95±2.25	16.22±3.87	20.03±6.01	6.06±1.29	8.86±1.74
FPGS1	7.05±0.35**	2.40±0.32**	3.97±0.32*	7.20±1.83*	9.43±3.61	7.37±1.36
FPGS2	35.29±1.46**	46.43±2.97**	34.96±6.70	39.21±10.93	6.99±3.21	6.84±2.20
GGH	10.18±2.02**	20.82±3.68**	8.25±1.27*	15.53±3.43*	2.67±1.40	5.14±1.90
GCSP	32.94±2.32	20.59±3.39	18.63±3.28	29.94±0.92	0.79±0.29	3.08±0.48
GCST	10.15±0.69	9.51±1.52	8.74±1.63	8.83±1.08	2.43±0.27	2.87±0.22
GCSH2	62.70±6.70	61.08±7.50	77.92±9.45	64.71±10.62	10.36±10.12	10.91±3.06
SHMT-GLY1	66.25±4.43	83.02±17.49	98.71±4.32	79.73±13.00	14.09±3.03*	21.58±2.08*
SHMT7-1	8.15±1.49*	5.04±1.26*	8.72±0.78	7.70±0.71	12.77±3.44*	4.09±0.40*
SHMT7-2	22.84±1.40	24.62±2.82	26.81±3.19*	18.55±2.20*	16.62±6.01*	4.90±0.74*
MTHFR1	42.65±3.50	38.89±7.28	46.46±3.05	66.41±17.36	5.32±1.62	2.71±0.31
MS1	0.19±0.1*	2.91±0.68*	0.84±0.35	0.71±0.11	3.18±2.80	2.08±0.23
MS2	71.04±7.90	53.13±10.36	70.30±5.78	78.50±19.02	1.53±0.51*	2.58±0.23*
DHC1	12.52±1.42	10.65±0.73	16.27±1.06	15.85±1.99	16.33±5.49	20.03±1.41
DHC2	38.73±1.28*	60.69±7.93*	58.62±4.45	102.51±20.11	64.53±9.72	81.86±10.98
DHC3	23.24±1.67*	15.64±2.94*	25.44±1.97	21.67±3.27	7.33±0.69	8.89±2.25

*: T-test value less than 0.05; **: T-test value less than 0.01.

Table S6. DEGs related to folate metabolism in *ppdk* mutant and wild type (from the published transcriptome data of Zhang *et al*, 2018) [67].

Gene	ID	HL_WT_Base	HL_homo_Base	Ratio of homo/WT	HL_WT_4cm	HL_homo_4cm	Ratio of homo/WT
ADCL2	Zm00001d039264	0.42	0.24	0.57	0.21	0.13	0.62
GCH-1	Zm00001d001959	0.72	0.81	1.13	0.78	2.12	2.72

DHNA1	Zm00001d031979	0.56	0.37	0.66	0.18	0.49	2.72
DHNA2	Zm00001d031995	0.41	0.59	1.44	0.28	0.47	1.68
GCSP	Zm00001d023437	4.14	4.59	1.11	17.07	14.17	0.83
GCST	Zm00001d002258	9.44	9.86	1.04	21.58	4.51	0.21
GCSH1	Zm00001d023699	2.98	5.47	1.84	19.85	11.54	0.58
SHMT-GLY1	Zm00001d049234	9.97	8.52	0.85	3.11	1.46	0.47
SHMT7-1	Zm00001d042661	0.31	0.48	1.55	0.37	1.31	3.54

HL, hight light; WT, wild type; homo, homozygous mutant; Tissue was collected from leaf three at 9 DAP 3 h into the light period from sections: base (1 cm above the leaf three ligule); 4 cm (4 cm above the leaf two ligule); RPKM (reads per kilobase per million mapped reads) was used to estimate the expression level of individual genes.

Table S7. FPKM of genes related to folate metabolism in *o2o2o16o16* mutant and the wild type (from ‘the published transcriptome data from Wang *et al*, 2019) [68].

Gene	ID	CML530_18DAP_A	CML530_18DAP_B	QCL8011_2_18DAP_A	QCL8011_2_18DAP_B	Ratio of Average QCL8011/CML530
ADCS	Zm00001d036484	3.23	3.8	1.38*	1.03*	0.35
ADCL1	Zm00001d029231	11.88	12.97	8.31*	5.83*	0.57
GCH-2	Zm00001d026531	1.04	0.92	2.27**	2.03**	2.20
DHNA2	Zm00001d031995	7.96	7.54	32.63	25.79	3.77
HPPK/DHPS	Zm00001d021338	10.14	11.81	4.12**	2.83**	0.32
DHFS1	Zm00001d023817	2.78	2.72	5.52**	5.4**	1.99
DHFS2	Zm00001d041625	4.33	4.78	8.49*	7.1*	1.72
DRTS4	Zm00001d049188	14.63	16.45	6.3**	5.83**	0.39
GGH	Zm00001d010744	30.41	33.03	11.71	17.03	0.45
GCSP	Zm00001d023437	25.1	23.3	6.37**	6.63**	0.27
GCST	Zm00001d002258	16.98	17.92	23.92**	23.85**	1.37
GCSH2	Zm00001d015378	51.54	49.76	95.06	123.71	2.16
GCSH3	Zm00001d053800	54.67	51.8	238.49**	261.89**	4.70
GCSH4	Zm00001d048974	0.34	0.47	4.09**	5.43**	12.06
SHMT-GLY1	Zm00001d049234	126.9	122.74	191.25**	198.73**	1.56
SHMT7-1	Zm00001d042661	10.84	12.34	2.48**	1.75**	0.18
SHMT7-2	Zm00001d012247	12.16	14.26	3.49**	2.89**	0.24
MS1	Zm00001d031128	287.42	291.19	326.88	363.5	1.19
MS3	Zm00001d033480	18.91	19.93	24.75**	25.91**	1.31
DHC1	Zm00001d005614	6.5	7.89	17.57**	20.43**	2.67
DHC2	Zm00001d010867	33.38	33.2	83.95*	61.97*	2.19
DHC3	Zm00001d053900	17.11	17.38	33.64**	38.66**	2.10
FTHS	Zm00001d020669	43.31	45.36	51.63*	51.24*	0.35
10-FDF	Zm00001d048550	2.09	2.37	0.75*	0.19*	0.57

CML530, wild type; QCL8011, mutant *o2o2o16o16*; 18DAP, 18 days after pollination; A,B, two replicates; FPKM (fragments per kilobase per million mapped reads) was used to estimate the expression level of individual genes. *, t-test value less than 0.05; **, t-test value less than 0.01.

Table S8. Primers used for qPCR.

Gene Abbreviation	Forward Primer Sequences (5′-3′)	Reverse Primer Sequences (5′-3′)
GAPDH	CCCTTCATCACCACGGACTAC	AACCTTCTTGGCACCACCCT
DHNA2	CGCCTGGATAGACCTCGC	GAGGCTTGCCAACCTTCACT
ADCS	CTTGTGAGTCAGATGATAGCCGAG	AATCTGTCTTCCGTGATGAGTAGC
DRTS-3	CATGTTCGAGCACTGGAGGAGC	CATCTCTATCTTCTGGTGGGGGTC
FPGS1	GAGAGGCAAGGCCATTTGAACAG	GTAGCGTGGGCAAACCATCTTGCAC
GGH	CAACGAGCCCGAAGACCGC	GCTCAAAGCCAAGACATTGGGC
SHMT7-1	GCGACATGGCACATATCAGTGGG	GCCAGCAATATGATTATTATGGGGCC
SHMT-GLY1	CTGCTCTGCGACATGGCGCAC	CATGCCAGCCCTTGGCCCTC
MS1	GATCCAGGACACCACCCAGATC	CTCCTCGGAAACGAAAAAGGC
